# Capacity to Consent in Healthcare: A Systematic Review and Meta-Analysis Comparing Patients with Bipolar Disorders and Schizophrenia Spectrum Disorders

**DOI:** 10.3390/medicina60050764

**Published:** 2024-05-05

**Authors:** Donato Morena, Matteo Lippi, Nicola Di Fazio, Giuseppe Delogu, Raffaella Rinaldi, Paola Frati, Vittorio Fineschi

**Affiliations:** Department of Anatomical, Histological, Forensic and Orthopedic Sciences, Sapienza University of Rome, 00185 Rome, Italy; donato.morena@uniroma1.it (D.M.); matteo.lippi@uniroma1.it (M.L.); nicola.difazio@uniroma1.it (N.D.F.); giuseppe.delogu@uniroma1.it (G.D.); raffaella.rinaldi@uniroma1.it (R.R.); vittorio.fineschi@uniroma1.it (V.F.)

**Keywords:** capacity to consent, mental competency, informed consent, schizophrenia spectrum and other psychotic disorders, bipolar and related disorders, systematic review, meta-analysis

## Abstract

*Background*: Mental capacity is a fundamental aspect that enables patients to fully participate in various healthcare procedures. To assist healthcare professionals (HCPs) in assessing patients’ capacity, especially in the mental health field, several standardized tools have been developed. These tools include the MacArthur Competence Assessment Tool for Treatment (MacCAT-T), the MacArthur Competence Assessment Tool for Clinical Research (MacCAT-CR), and the Competence Assessment Tool for Psychiatric Advance Directives (CAT-PAD). The core dimensions explored by these tools include Understanding, Appreciation, Reasoning, and Expression of a choice. *Objective*: This meta-analysis aimed to investigate potential differences in decision-making capacity within the healthcare context among groups of patients with bipolar disorders (BD) and schizophrenia spectrum disorders (SSD). *Methods*: A systematic search was conducted on Medline/Pubmed, and Scopus. Additionally, Google Scholar was manually inspected, and a manual search of emerging reviews and reference lists of the retrieved papers was performed. Eligible studies were specifically cross-sectional, utilizing standardized assessment tools, and involving patients diagnosed with BD and SSD. Data from the studies were independently extracted and pooled using random-effect models. Hedges’ *g* was used as a measure for outcomes. *Results*: Six studies were identified, with three studies using the MacCAT-CR, two studies the MacCAT-T, and one the CAT-PAD. The participants included 189 individuals with BD and 324 individuals with SSD. The meta-analysis revealed that patients with BD performed slightly better compared to patients with SSD, with the difference being statistically significant in the domain of Appreciation (ES = 0.23, 95% CI: 0.01 to 0.04, *p* = 0.037). There was no statistically significant difference between the two groups for Understanding (ES = 0.09, 95% CI:−0.10 to 0.27, *p* = 0.352), Reasoning (ES = 0.18, 95% CI: −0.12 to 0.47, *p* = 0.074), and Expression of a choice (ES = 0.23, 95% CI: −0.01 to 0.48, *p* = 0.60). In the sensitivity analysis, furthermore, when considering only studies involving patients in symptomatic remission, the difference for Appreciation also resulted in non-significant (ES = 0.21, 95% CI: −0.04 to 0.46, *p* = 0.102). *Conclusions*: These findings indicate that there are no significant differences between patients with BD and SSD during remission phases, while differences are minimal during acute phases. The usefulness of standardized assessment of capacity at any stage of the illness should be considered, both for diagnostic-therapeutic phases and for research and advance directives. Further studies are necessary to understand the reasons for the overlap in capacity between the two diagnostic categories compared in this study.

## 1. Introduction

The mental capacity constitutes a core element to enable the full participation of patients in their clinical pathways and the consolidation of the therapeutic alliance between them and healthcare professionals (HCPs) [1].

Its foundations are based on cognitive autonomy and the integrity of decision-making abilities, which collectively allow patients to make free, conscious, and informed choices [2].

In the healthcare context, mental capacity is primarily based on the competence to complete informed consent regarding diagnostic and therapeutic procedures, to participate in research, and to establish advanced directives [3].

The difficulties associated with judgments on mental capacity determined solely by clinical assessments [4] have led to the development of various standardized assessment tools over the years [5].

In mental health, instruments have been developed for the assessment of treatment consent (Grisso et al., 1997), research participation [6,7], and advanced directives [8].

Most of these tools consist of subscales for assessing the four fundamental dimensions that have been identified as determinants of decisional capacity [9], namely the abilities to: (i) comprehend the pertinent components of one’s medical condition and assimilate all information relevant to decisions (Understanding); (ii) utilize this information in evaluating its implications in alignment with personal values, beliefs, and expectations, including an assessment of potential consequences (Appreciation); analyze available information by structuring it in a logical and rational sequence, involving the evaluation of pros and cons, and assess potential therapeutic alternatives (Reasoning); communicate a decision or identify a designated individual who can assist in making the most suitable decision (Expression of a choice).

Mental capacity is a specific and dynamic ability. Therefore, HCPs should be mindful that assessing capacity cannot be considered a ‘global’ evaluation of patients’ overall mental status or their ability to make various decisions.

Instead, it should focus on a specific decision-making task at a particular moment in time only [10,11], while medical procedures requiring consent over extended periods necessitate recurrent assessments [12].

Changes in mental capacity may result from physiological processes, such as aging [13], or they could be influenced by multifaceted factors in pathological conditions, as observed in serious mental illnesses (SMI) [14].

A meta-review of literature reviews evaluating the capacity of patients with SMI to make decisions about their healthcare concluded that the majority are competent in making appropriate decisions in this regard [15].

However, this study did not explore potential variances among specific types of psychiatric disorders. Specifically, reviews exclusively focused on bipolar disorders were absent, with the majority pertaining to cohorts characterized by heterogeneous diagnoses or schizophrenia.

Moreover, a broad variability among the scrutinized studies emerged in assessing full mental capacity, particularly when considering all four components it encompasses. This heterogeneity can be explained not only by differences among specific disorders but also by factors such as the phase of illness and setting, as well as potentially varied socio-demographic and neuropsychological characteristics across studies.

It should be considered that certain symptomatic conditions, such as those occurring during the acute phases of affective or psychotic disorders, have the potential to hinder the competence to consent.

This may also be exacerbated by exposure to stressful situations, such as hospitalization, or the administration of higher medication doses [16].

However, for individuals with SMI, even the stable phases can be characterized by mental incapacity, mainly due to cognitive impairment that affects the ability to concentrate, understand, assimilate information, and maintain consistency in decision-making [17].

Specifically, for patients with schizophrenia and bipolar disorders, psychopathological status, insight, and cognitive performance can prove pivotal in influencing their decision-making processes [18,19,20].

Regarding clinically stable outpatients with schizophrenia, some studies have found an overlap with the competence to consent to treatment or hospitalization when compared to the general population [16,18].

Nevertheless, a meta-analysis conducted by Wang et al. [21] demonstrated that particularly in the elderly, individuals with schizophrenia or schizoaffective disorder were significantly more prone to experiencing impaired decision-making capacity across all four core domains of competence when compared to healthy controls. Moreover, this was evident both for clinical research and treatment consent.

For these patients, additional distinctions have been observed in the ability to give consent for participation in research programs compared to treatment, particularly during acute phases of the illness, which are present in only approximately half and one-third of the sample, respectively [22].

Hostiuc et al. [23] in their meta-analysis further highlighted differences between groups of patients with schizophrenia and healthy controls, emphasizing the necessity of utilizing enhanced informed consent forms when including such patients in clinical trials.

Differently from patients with schizophrenia, the available data for patients with bipolar disorders is very limited, as well as conflicting, and inconclusive [24,25].

Palmer et al. compared the decisional capacity of three groups: one consisting of outpatients with bipolar disorders, another with schizophrenia, and a third comprising healthy subjects [26]. Both groups of patients exhibited a current minimal psychopathological status. There were no differences between the group of patients with schizophrenia and bipolar disorders in all dimensions of capacity. The group of healthy subjects reported significantly higher scores in Understanding compared to both patient groups, and in Appreciation exclusively compared to the group of patients with schizophrenia. There were no significant correlations between scores of manic symptomatology and competence dimensions, while depressive symptomatology showed a negative correlation with the Reasoning score.

The study by Klein et al. [25] involving patients with bipolar disorders highlighted a correlation between the severity of psychotic symptomatology and poorer performance on the Understanding and Appreciation dimensions. Instead, there was a reverse correlation between depressive symptoms and scores in Understanding, Appreciation, and Reasoning.

However, it is worth noting that this data remains somewhat controversial [27,28].

Koukopoulos et al. [29] found that patients hospitalized for a manic/hypomanic episode scored worse than outpatients in Understanding, Reasoning, and Expressing a choice, but not in Appreciation. Outpatients in a phase of clinical stability were more capable in the dimension of Understanding the characteristics of an alternative advance treatment decision. General cognitive functioning positively correlated with scores in all four dimensions of competence, whereas manic symptomatology showed an inverse correlation and depressive symptomatology correlated only with Appreciation scores.

Despite these studies, as mentioned, the existing literature on the field of mental capacity presents a limited amount of evidence, especially concerning bipolar disorders.

Given the paucity of data for this patient group and the absence of comparisons with patients with schizophrenia spectrum disorders (primarily schizophrenia or schizoaffective disorder), which are the main diagnoses within the SMI category, this study aimed to assess differences in decision-making capacity between these two patient groups.

This approach could potentially broaden the existing research evidence for patients with bipolar disorders by leveraging findings from studies on schizophrenia spectrum disorders.

Additionally, if differences are identified, it may suggest that phase-related factors, primarily associated with psychopathological status, influence decision-making capacity.

However, in the absence of differences, particularly during clinical remission, as per our hypothesis, cognitive factors may be considered dominant and potentially compromised in both groups.

## 2. Materials and Methods

Our quantitative systematic review was conducted following the Preferred Reporting Items for Systematic Reviews and Meta-Analyses (PRISMA) 2020 Guidelines [30,31].

### 2.1. Literature Search

**Figure 1:**
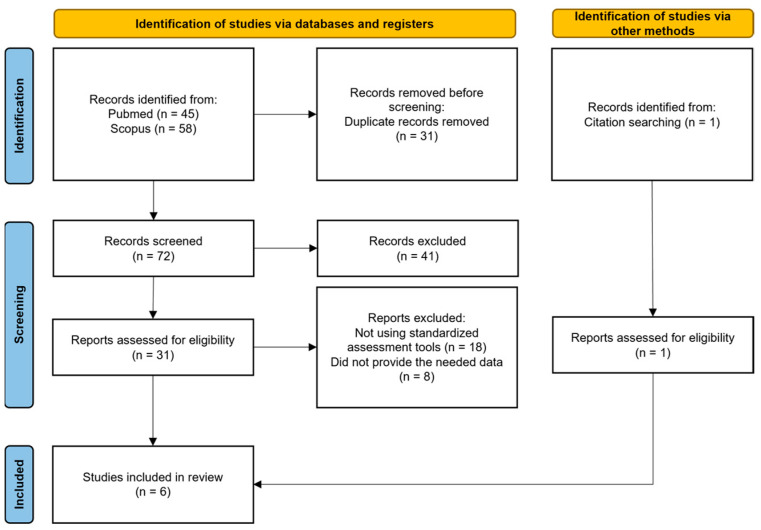
Our review strategy following PRISMA standards.

The process of identifying eligible studies for the systematic review and meta-analysis is shown in is outlined in Figure 1.

Potential articles used in the meta-analysis were identified from Scopus, Pubmed, and Google Scholar. No temporal filters were applied. For the search on Scopus, the keywords used were “TITLE-ABS-KEY-AUTH (schizhophreni*) AND/OR TITLE-ABS-KEY-AUTH (bipolar*) AND TITLE-ABS-KEY-AUTH (competen*) AND TITLE-ABS-KEY-AUTH (consen*)”. For the search on PubMed, the keywords used were “((schizhophreni*) AND/OR (bipolar*)) AND (competen*) AND (consen*) any field”. Emerging reviews and reference lists of the retrieved papers were also manually searched by two investigators (D.M. and M.L.). Google Scholar was manually inspected, specifically looking for studies utilizing standardized tools for the assessment of capacity (e.g., MacCAT-T, MacCAT-CR, SICIATRI, SICIATRI-R, CAT-PAD).

Initially, eligibility screening was conducted on the abstracts of papers identified through the described procedures. Papers that successfully underwent this screening process were subsequently subjected to a more comprehensive assessment for potential inclusion in our study, involving a thorough examination of the full text. Two independent reviewers (D.M. and M.L.) evaluated the reports and extracted data; any disagreements were resolved by a third author (either P.F. or V.F.).

A comprehensive approach, deliberately keeping eligibility criteria broad, was adopted. The inclusion criteria specifically targeted cross-sectional studies where standardized tools were utilized to measure the capacity to provide consent, involving patients diagnosed with bipolar disorders and schizophrenia spectrum disorders.

Papers not written in English or not published in peer-reviewed journals were excluded.

The protocol for this review has been registered in the International Prospective Register of Systematic Reviews (PROSPERO registration number CRD42024502141).

### 2.2. Data Extraction

A standardized form was employed to extract data from the included studies, aiding in study quality and evidence synthesis. Extracted information encompassed the study’s focus; participant characteristics such as age, sex (expressed as percentages of female participants), years of education, diagnosis, stage (acute vs. chronic), and duration of the illness; baseline symptom severity; the type of assessment tools used to determine the capacity to provide consent; and the information required for the assessment of the risk of bias. Extraction was independently conducted by two reviewers (D.M. and M.L.) in duplicate. A third reviewer (V.F.) was consulted when needed.

### 2.3. Quality Assessment

The quality of the included studies was assessed by two independent reviewers (D.M. and M.L.) using the Newcastle-Ottawa Scale adapted for cross-sectional studies [32,33]. This scale assigns a maximum of 10 “stars” for the lowest risk of bias. Three areas are explored: (1) study sample selection (5 stars); (2) comparability of groups (2 stars); (3) outcomes (3 stars). Any disagreements were resolved through comparison between the two reviewers. Four studies scored ≥ 7 stars, indicating good quality [34,35,36,37], while two studies scored 6 stars, which still reflects satisfactory quality [8,26]. The results are summarized in Table 1.

### 2.4. Outcome Measures

Differences between patients diagnosed with bipolar disorders and schizophrenia spectrum disorders were investigated concerning the main domains constituting the competence assessment scales, namely Understanding, Appreciation, Reasoning, and Expression of a choice. These outcomes were further explored through metaregressions and subgroup and sensitivity analyses.

Regarding general psychopathology, in cases where studies reported multiple rating instruments for symptoms, only one scale per study was selected. Priority was given to the Brief Psychiatric Rating Scale (BPRS) [38].

### 2.5. Meta-Analysis Procedure

We conducted four meta-analyses to examine the differences between the two studied groups of patients with bipolar disorders (hereafter referred to as the ‘BD group’) and schizophrenia spectrum disorders (hereafter referred to as the ‘SZ group’) in the four dimensions considered by the literature as constitutive of decisional capacity in the healthcare field, namely Understanding, Appreciation, Reasoning, and Expression of a choice.

Effect sizes were computed utilizing means and standard deviations (SD). Since scores on the decisional capacity subscales were continuous data obtained from different scales (i.e., CAT-PAD, MacCAT-T, MacCAT-CR), but mostly investigated similar domains (i.e., understanding, appreciation, reasoning), and given the small sample size of patients found in the selected studies, Hedges’s *g* with 95% confidence intervals (CIs) was chosen to analyze the studies [39].

The mean effect size for the group of studies was calculated by pooling individual effect sizes using a random-effect model instead of a fixed-effect model, given that the selected studies were not identical (i.e., did not have either an identical design or target the same population).

Values of 0.20, 0.50, and 0.80 for Hedges’ *g* were considered indicative of small, medium, and large effects, respectively [40].

Heterogeneity among studies in each meta-analysis was assessed using the chi-squared statistic (Q), I^2^, and Tau^2^. Substantial heterogeneity was considered if I^2^ exceeded 30%, and either Tau^2^ was greater than zero or there was a low p-value (less than 0.10) in the chi-squared statistic (Q) test for heterogeneity. I^2^ measures the proportion of heterogeneity to the total observed dispersion and is not influenced by low statistical power. We considered I^2^ values as low ranging from 0% to 25%, intermediate from 25% to 50%, moderate from 50% to 75%, and high when ≥75% [39]. A subgroup analysis was performed on studies sharing the same decisional capacity instrument and the same psychological status of stable conditions. We deemed *p* < 0.05 (two-tailed) as statistically significant. The risk of publication bias was evaluated through a visual examination of funnel plots and a statistical test of asymmetry (Egger test) [41].

The meta-analysis was carried out using the software ProMeta 3.

## 3. Results

We found 31 potentially eligible studies from 103 records obtained from the selected databases and 1 after references screening. After reviewing the full content of the papers, 26 papers were excluded for various reasons: 18 did not examine the capacity to consent to treatment or clinical research, and 8 did not supply the needed data. Regarding the exclusion of these articles, we specify that initially, we identified 9 articles worthy of inclusion in our meta-analysis as they provided an assessment of competence to consent in both patients diagnosed with bipolar disorders and schizophrenia spectrum disorders. However, the available information within the articles themselves did not initially allow for obtaining the necessary data for our study. For this reason, personal communication was sent individually to the corresponding authors of each article, along with a specially designed data sheet for each study, with the aim of collecting the required data. Only one author (Prof. Hotopf) kindly responded and made the requested data available [35]. Therefore, out of these 9 articles, we were forced to exclude 8, while only 1 was included (for the list of studies not included see Supplementary Material ‘Supplement S1’).

Finally, 6 articles were included in our meta-analysis. All of them compared the domains of Understanding, Appreciation, and Reasoning, while only three reported the results of the Expression of a choice.

### 3.1. Studies, Participants, and Treatment Characteristics

All six studies selected in this meta-analysis have been published in peer-reviewed journals and were all conducted at the national level: three in the USA [8,26,34], one in England [35], one in Italy [37], and one in Colombia [36]. The majority of these (four) were conducted at a multicenter level [8,34,36,37]. Furthermore, all the studies included had a cross-sectional design. In addition, two studies aimed to validate a psychometric scale: López-Jaramillo et al. [36] intended to validate and adapt the MacCAT-CR scale to the Spanish language, while Srebnik et al. [8] aimed to validate a new instrument for assessing the decision-making capacity of psychiatric patients (CAT-PAD).

Regarding the recruitment of participants, four studies recruited patients anew [8,34,35,37], while two studies enrolled participants from those already recruited for two larger studies [26,36].

Additionally, two studies recruited patients from inpatient psychiatric wards [35,37], while the other four studies recruited participants from outpatient settings, specifically from community mental health centers [8,26,34,36]. The characteristics of the included studies are summarized in Table 2.

Among the six studies, as assessment tools, one utilized the CAT-PAD (Srebnik et al., 2004), while three studies employed the MacCAT-T [34,35,37] and two the MacCAT-CR [26,36].

The MacCAT-T is a semi-structured interview designed to assess key aspects of treatment-related decision-making, aligning with commonly applied legal standards for competence to consent to treatment [42].

The subscales within the MacCAT-T evaluate understanding, which involves grasping information about the disorder and the main features of the treatment, as well as presumed associated risks and benefits (rated 0–6); appreciation, reflecting patients’ ability to comprehend their own diagnosis and treatment (rated 0–4); reasoning ability, encompassing consequential and comparative thinking, and logical consistency (rated 0–8); and the ability to clearly express a choice (rated 0–2).

The MacCAT-CR is a semi-structured interview that utilizes the same multidimensional capacity model as MacCAT-T but includes 21 items assessing the well-known four abilities related to competence, specifically in the context of consent to clinical research: understanding of purposes, procedures, potential benefits, risks, of the research project (rated 0–26); appreciation of the impact of participation in research on personal condition (rated 0–6); reasoning about the consequences of participation (range 0–8); and consistent expression of a choice (rated 0–2).

The Competence Assessment Tool for Psychiatric Advance Directives (CAT-PAD) is a tool designed to assess competence in completing a psychiatric advance directive (PAD). Despite some differences from the Mac-CAT scales, it similarly involves a series of disclosures of information representative of what is relevant for decisions about completing a PAD [8]. The CAT-PAD consists of three subscales: understanding (rated 0–20), appreciation (rated 0–6), and reasoning (rated 0–10), totaling 18 items. Its construct is similar to the MacCAT-T Alternative Treatment (AT) variant, which measures the ability to make a valid choice between two proposed alternative treatments, including the current one, in case of a possible future acute phase of illness [29].

In the overall analysis, a total of 189 patients were included for the ‘BD group’ and 324 for the ‘SZ group’ for the dimensions of Understanding, Appreciation, and Reasoning, while for the dimension of Expression of choice, which was investigated by only three studies [26,35,37], 107 and 180 patients were respectively included.

More information about the included studies is listed in Table 3.

### 3.2. Competence to Consent

The results are presented in alignment with evidence demonstrating the existence of four fundamental domains crucial for decision-making capacity, which are also mirrored in the primary assessment scales: Understanding, Appreciation, Reasoning, and Expression of choice.

#### 3.2.1. Understanding

For Understanding (Table 4), the ‘BD group’ exhibited a slightly positive effect size (ES = 0.09) but was not statistically significant (*p* = 0.352). Despite some discrepancies in the direction of the effect sizes, there was no significant heterogeneity (Q (5) = 4.21, *p* = 0.519).

#### 3.2.2. Appreciation

As seen in Table 5, a small but significant effect size was obtained for Appreciation, with the ‘BD group’ performing slightly better than the ‘SZ group’ (ES = 0.23, *p* = 0.037). Also for this dimension, there was no significant heterogeneity among the studies (Q(5) = 6.73, *p* = 0.242).

The worst results for the ‘BD group’ were obtained in the study by Cairns et al. [35], with an ES = −0.04 (*p* = 0.840). Anyway, this study included patients in an acute phase of psychopathological symptoms and with a particularly high severity index of illness (BPRS: ‘SZ group’ = 48.3 ± 10.6; ‘BD group’ = 46.8 ± 8.0).

A null effect size was also reported for the study by López-Jaramillo et al. [36]. In this study, the compared groups had significantly different mean ages (*p* < 0.001), with the ‘BD group’ (46.3 y ± 12.4) being older than the ‘SZ group’ (34.9 y ± 10.5).

Due to the limited availability of data, additional analysis was unable to identify which clinical, demographic, and illness-related variables were relevant in differentiating the ‘BP group’ and ‘SZ’ concerning this dimension of capacity.

#### 3.2.3. Reasoning

For Reasoning (Table 6), the ‘BD group’ had a slightly positive effect size (ES = 0.18) but was not statistically significant (*p* = 0.236). The only study with a significant effect size (*p* = 0.001) was that of Mandarelli et al. [37], with an ES = 0.65. In this case, the studies exhibited significant heterogeneity (Q(5) = 12.40, *p* = 0.030).

The only study to highlight a negative effect size (ES = −0.31, *p* = 0.21) was that of Appelbaum & Redlich [34]. However, the results of this study showed a discrepancy between the data collected at one recruitment center (Durham), where the results of the two groups tended to overlap (SZ = 6.94 ± 1.61; BD = 6.86 ± 1.46), compared to another center (Worcester) (SZ = 5.20 ± 1.42; BD = 4.93 ± 1.94).

The only study to show a negative effect size (ES = −0.31, *p* = 0.21) was that of Appelbaum & Redlich [34]. However, the statistical analysis conducted by the researchers did not reveal any significant difference between the scores of the ‘BD group’ compared to that of the ‘SZ group’.

#### 3.2.4. Expression of a Choice

For the Expression of a choice (Table 7), the BD group had a slightly positive effect size (ES = 0.23) but not significant (*p* = 0.060). None of the studies had a significant effect size, and overall, they were not heterogeneous (Q(3) = 1.80, *p* = 0.407).

### 3.3. Sensitivity

To address the challenges associated with comparing studies utilizing various decisional capacity measures, we conducted a subgroup analysis involving 169 patients from the ‘BD group’ and 283 from the ‘SZ group’ across five studies. These studies employed similar instruments, such as MacCAT-T and Mac-CAT-CR. Notably, we excluded the study conducted by Srenbnik et al. [8], where the CAT-PAD scale was utilized. Our analysis focused on evaluating scores related to Understanding, Appreciating, and Reasoning (refer to Supplementary Material ‘Table S2’ for details).

We did not conduct an analysis with the Expression of choice subscale, as the studies included [26,35,37] were identical in the overall analysis.

Utilizing random-effect models, the ‘BD group’ did not demonstrate significant differences compared to the ‘SZ group’ across any of the investigated competence dimensions. Specifically, for Understanding (ES = 0.04, 95% CI: -0.16 to 0.23, *p* = 0.707; Q (4) = 2.08, I^2^ = 0.00, Tau^2^ = 0.00), Appreciation (ES = 0.21, 95% CI: -0.04 to 0.46, *p* = 0.102; Q (4) = 6.50, *p* = 0.165, I^2^ = 38.43%, Tau^2^ = 0.03), and Reasoning (ES = 0.12, 95% CI: −0.21 to 0.45, *p* = 0.465, Q (4) = 11.05, *p* = 0.465, I^2^ = 63.81%, Tau^2^ = 0.09).

A subsequent subgroup analysis, including studies with patients in a clinical remission status, was conducted to minimize potential interference related to the acute psychopathological phase of illness. Hence, two studies, which recruited patients consecutively admitted to adult psychiatry wards [35,37] were excluded (refer to Supplementary Material ‘Table S3’ for details).

For this analysis as well, using random-effect models, the 113 patients of ‘BD group’ did not exhibit significant differences compared to the 175 patients of ‘SZ group’ across any of the investigated competence dimensions, that is Understanding (ES = 0.15, 95% CI: −0.10 to 0.41, *p* = 0.226; Q (3) = 3.24, *p* = 0.356, I^2^ = 7.37, Tau^2^ = 0.00), Appreciation (ES = 0.21, 95% CI: −0.04 to 0.46, *p* = 0.095; Q (3) = 3.17, *p* = 0.367, I^2^ = 5.27%, Tau^2^ = 0.00), Reasoning (ES = 0.08, 95% CI: −0.24 to 0.39, *p* = 0.632; Q (3) = 4.98, *p* = 0.173, I^2^ = 39.79%, Tau^2^ = 0.04).

For the Expression of a choice, only the study conducted by Palmer et al. [26] remained, comparing 31 patients of the ‘BD group’ to 31 patients of the ‘SZ group’, and indicating no significant differences (ES = 0.35, *p* = 0.167, 95% CI: −0.15 to 0.84).

### 3.4. Publication Bias and Sensitivity Analysis

Visual inspection of the funnel plot and Egger’s regression test did not show any publication bias for the subscales of Understanding (t = 1.27; *p* = 0.272), Appreciation (t = 0.03; *p* = 0.976), Reasoning (t = −0.64; *p* = 0.559), and Expression of a choice (t = −0.01; *p* = 0.992) in the comparison between ‘BD group’ and ‘SZ group’. The application of the trim-and-fill method, revealing symmetrical funnel plots for all four subscales of capacity, further suggested the consistency of the results. However, caution is warranted when excluding the presence of publication bias, given the limited statistical power of the test in a meta-analysis with a small number of trials [39,41].

## 4. Discussion

In our study, the primary objective was to investigate potential differences in decision-making capacity within the healthcare context among groups of patients with bipolar disorders and schizophrenia spectrum disorders (primarily schizophrenia or schizoaffective disorder). This addresses the existing gap in evidence in this field specifically for patients with bipolar disorders.

In other words, given the existence of data on mental capacity mostly for patients with schizophrenia spectrum disorders [43], we endeavored to comprehensively analyze studies comparing these patients with those diagnosed with bipolar disorders, to assess whether the same evidence could be applied to the latter.

Moreover, identifying any general similarities or differences, as well as differences in specific dimensions of mental capacity, was also useful for developing etiological hypotheses and suggesting research focuses for further studies.

The results of our meta-analysis, conducted on all available studies comparing the two groups of patients, revealed no significant differences in the dimensions of Understanding, Reasoning, and Expression of a choice.

Conversely, in terms of Appreciation, the ‘BD group’ achieved a slightly higher score compared to the ‘SZ group’, reaching statistical significance.

This domain is crucial to ensure that patients not only comprehend the general medical information (covered by Understanding) but also perceive the applicability and relevance of this information within the context of their own health status and personal circumstances.

However, it’s important to note that in the sensitivity analysis, which exclusively considered studies involving patients in stable clinical conditions and excluded those recruiting patients in acute phases (such as those hospitalized in emergency psychiatric wards), the difference in Appreciation also proved to be non-significant.

To understand the reasons behind this evidence, we conducted an in-depth examination of the two studies excluded from the sensitivity analysis due to the involvement of patients in an acute state.

Peculiarly, the study by Mandarelli et al. [37] revealed the presence of psychopathological heterogeneity between the ‘BD’ and ‘SZ’ groups, with significant differences observed for some scores on the utilized assessment scale, the BPRS.

The ‘BD group’ exhibited significantly higher scores for the excitement subscale, while the ‘SZ group’ showed significantly higher scores for the positive and negative symptoms subscales.

Therefore, we hypothesize that, in this study, the prevalent psychotic symptomatology in the ‘SZ group’ may have negatively influenced the Appreciation, thus causing a significant divergence between the two groups of involved patients.

It is possible that psychotic symptomatology may have a greater impact on this dimension of the MacCAT-T even compared to excitatory symptomatology.

However, this point remains controversial: for example, Owen et al. [44] found an association between the lack of capacity measured with the MacCAT-T and manic episodes.

The absence of additional information on possible concurrent psychotic symptomatology (which may be present during manic episodes) determines difficulties in inferring whether the impact on capacity was predominantly due to psychotic or excitatory symptomatology.

Conflicting data are also reported regarding the correlation between Appreciation and negative symptomatology [42,45].

Positive symptomatology, on the other hand, has a well-known detrimental impact on Appreciation [26]. This evidence could be mediated by the effect of psychotic symptoms on insight, as a negative correlation has been found between the latter and Appreciation and Reasoning [46]. The dearth of data, however, has prevented the verification of this point in our analysis.

However, an interesting finding is represented by the absence of significant differences in Appreciation during stable phases of illnesses, suggesting that other elements may be at play, shared by both groups of patients.

Overall, under stable conditions, despite trends favoring the ‘BD group’ in every dimension of capacity, no significant differences emerged in our meta-analysis.

This data is quite surprising when considering that historically, the group of patients with bipolar disorders is believed to be more suitable for a *restitutio ad integrum* during symptomatic remission [47].

On this point, it is important to highlight some evidence from recent research that has compared the two groups considered in the present study.

Similar to individuals diagnosed with schizophrenia spectrum disorders, it has been found that patients with bipolar disorders also suffer from poor overall functioning, contrary to historical assumptions [48]. This impairment persists even during phases of symptomatic remission [49], and, notably, the World Health Organization (WHO) has designated this category as the 12th leading cause of disability globally [50].

A hypothetical pivotal factor in explaining the similar results in functional outcomes, as well as the findings of our study on mental capacity, could be represented by cognitive functions.

Cognitive impairment is well acknowledged in both patient groups, albeit with qualitative and quantitative distinctions [51], and it could be particularly predominant compared to psychopathology during stable phases for mental capacity.

Studies employing comprehensive cognitive assessment batteries [14,18,52,53] have indicated a relationship between overall cognitive functioning and the capacity to provide consent.

Even though specific links between cognitive domains and the dimensions influencing mental capacity have not been demonstrated [54], it is conceivable that underlying deficit domains are shared among patients with schizophrenia spectrum disorders and bipolar disorders.

Moreover, it is worth noting that patients with bipolar disorders tend to exhibit cognitive trajectories that overlap with those of individuals with schizophrenia over time, while cognitive impairment emerges during early adulthood for the latter group [55].

This data could explain why, in the study by Mandarelli et al. [37], which assessed a relatively young sample of patients, significant differences between the two groups emerged for Appreciation and Reasoning.

A final point concerns the subscale Expression of a choice, for which literature does not report differences between groups of patients with schizophrenia or bipolar disorders compared to healthy subjects [18,26]. However, this subscale has been noted as the least sensitive as defined by current assessment tools.

## 5. Limits

For our work, several limitations should be recognized. Firstly, the sample size in the included studies was relatively small. We attempted to retrieve the data from an additional 8 plausible studies identified in the literature, but it was not possible to obtain them.

Secondly, diverse versions of the MacCAT were utilized across the studies, and one study employed a tool specifically designed for the assessment of advance directives. As a result, random-effects models were employed. Furthermore, there was no differentiation between bipolar I, bipolar II, and cyclothymic disorder, as the focus was on an overarching assessment of the bipolar spectrum [56]. Nevertheless, despite differences between these diagnoses, they are all characterized by “unusual shifts in mood, energy, activity levels, concentration and the ability to carry out day-to-day tasks” [57].

## 6. Conclusions

With our study, we aimed to gather evidence on the decision-making capacity of patients with bipolar disorders and schizophrenia spectrum disorders. We found this topic valuable as informed consent competency is often assessed merely on a clinical basis [58], and patients with bipolar disorders are typically considered more functional during remission phases compared to those with schizophrenia spectrum disorders. The results of our meta-analysis, however, indicate that there are no significant differences between the two groups, at least as revealed by standardized assessment tools. We hypothesize that cognitive aspects predominantly play a role in determining capacity during stable phases.

These results lead to the consideration that it is useful to assess the capacity to provide consent at any stage of illness, both for diagnostic-therapeutic phases and for research and advance directives.

However, in the clinical *milieu*, assessment of capacity should be always considered in a personalized manner, avoiding a general judgment of presence or absence. Assessment scales serve as a support to delve into the dimensions of competence and provide guidance to HCPs, in the absence of a specific cut-off to determine whether a person is legally competent or not.

Furthermore, HCPs should carefully consider the specificity and dynamic nature of mental capacity.

The results of our study provide a further indication regarding the limited reliability of assessments based on clinical judgment, especially relying solely on the value of the diagnosis.

It is emphasized that despite similar results emerging for both patient groups, additional elements are lacking to define the reasons for this overlap. Further studies could clarify this point by examining whether the absence of differences in capacity between the two patient groups is only based on cognitive profiles or if additional variables, potentially imbricated, have a peculiar role.

## Figures and Tables

**Table 1:** The Newcastle-Ottawa Quality Assessment Scale adapted for cross sectional studies.

Author	Selection	Comparability	Outcome (Total) ^#^
Srebnik et al., 2004 [8]	**	**	** (6)
Cairns et al., 2005 [35]	***	**	*** (8)
Appelbaum & Redlich 2006 [34]	***	**	*** (8)
Palmer et al., 2007 [26]	**	**	** (6)
López-Jaramillo et al., 2016 [36]	***	**	*** (8)
Mandarelli et al., 2018 [37]	***	**	*** (8)

^#^ (Total = sum of the stars of the three items ‘Selection’, ‘Comparability’ and ‘Outcome’; maximum score = 10); good studies: ≥7 stars; satisfactory studies: 5–6 stars; unsatisfactory studies: 0 to 4 stars.

**Table 2:** Characteristics of the included studies.

No.	Author, Year	Study Aim	Study Design	Inclusion Criteria	Exclusion Criteria	Sample Size	Sampling Technique	Tools	Key Findings
1	Srebnik et al., 2004 [8]	To validate a new instrument (CAT-PAD) designed to assess patients’ skills in evaluating their decision-making capacity regarding therapeutic choices related to their disorder	Validation study of a psychometric scale	- Age ≥ 18 years - A minimum of two psychiatric emergency room visits or hospitalizations within the past 2 years - Managed by community mental health centers - English speaking	NA	N = 80	Outpatient selected because at highest risk of experiencing crises where PADs could be utilized	- CAT-PAD - PSAS - PSS	- Based on the psychometric data, the findings indicated that the CAT-PAD is a suitable tool for evaluating the ability to complete a PAD - In post hoc regression modeling, individuals with schizophrenia exhibited markedly lower total CAT-PAD scores compared to the bipolar and depression groups
2	Cairns et al., 2005 [35]	To deduce the prevalence of psychiatric in-patients who lacked the mental capacity to make decisions about their ongoing treatment	Multicenter cross-sectional study	Admission to a psychiatric ward at one of the three hospitals actively involved in research	- Incapacity of giving informed consent for participation in a study - Not currently assuming routine psychoactive drugs - No English speaking	N = 112 (Consecutive patients admitted to three general adult psychiatric wards and invited to participate)	Reasonably representative of patients who need to be admitted to a psychiatric inpatient unit	- MacCAT–T - BPRS - SAI–E - MMSE - BPCS	- Out of 112 in-patients, 49 individuals (43.8%) lacked decisional capacity related to treatment - Incapacity was associated with mania, psychosis, and poor insight
3	Appelbaum & Redlich 2006 [34]	To determine decisional capacity in psychiatric patients subjected to leverage	Multicenter cross-sectional study	- Age: 18–65 years - At least one visit for outpatient care at a Community Mental Health Center within the last 6 months, and initial service contact at least 6 months before	No English speaking	N = 120 (enrolled for decisional capacity assessment as part of a larger study with 1011 participants)	Chosen to represent the most prevalent mental illness diagnoses and psychopharmacologic treatments used within the studied population	- MacCAT–T - BPRS - GAF - ITAQ	No significant or consistent connections were observed between decision-making capacity regarding treatment and the use of leverage to promote treatment adherence
4	Palmer et al., 2007 [26]	To assess the decisional capacity of bipolar patients vs. those with schizophrenia and healthy controls	Cross-sectional study	- Diagnosis of schizophrenia or bipolar disorder - Age ≥ 40 years	- -No English speaking - Substance use disorder or dementia in treatment with atypical antipsychotics	N = 90	Outpatients recruited from board-and-care facilities, day treatment programs, University and Veterans’ Affairs psychiatry services	- MacCAT-CR - PANSS - HAM-D - BIQ - WAIS	- Bipolar patients exhibited worse insight compared to healthy controls - The decisional capacity of bipolar patients was not found to be significantly different from patients with schizophrenia - Neurocognitive impairments and negative symptoms demonstrated a significant correlation with the extent of decisional capacity
5	López-Jaramillo et al., 2016 [36]	- To investigate the correlation between insight and the ability to provide consent to participate in research - Validation of MacCAT-CR	- Cross-sectional and longitudinal study - Validation study of a psychometric scale	- Age ≥ 18 years - Being physically capable of completing the study	No Spanish-speaking	N = 120	Voluntary enrollment of patients for any of the research studies conducted by the psychiatric research group from the university or the mood disorders program from a hospital facility	- MacCAT-CR - SAI-E	Subjects with a higher level of illness insight show a better ability to provide informed consent for research participation
6	Mandarelli et al., 2018 [37]	To assess the ability of involuntarily admitted patients to make treatment decisions and consent to psychiatric treatment	Multicenter, cross-sectional study	Consecutive recruitment of acute psychiatric patients hospitalized under involuntary admission	Refusing to participate overall	N = 13	Voluntary recruitment of subjects involuntarily hospitalized due to an acute mental disorder and the need for treatment	- MacCAT-T - BPRS - MMSE	The patients with bipolar disorders generally achieved higher scores than those with schizophrenia spectrum disorders in MacCAT-T Appreciation and Reasoning

**Abbreviations**: BIQ = Birchwood Insight Questionnaire; BPCS = Brief Perceived Coercion Scale; BPRS = Brief Psychiatric Rating Scale; CAT-PAD = Competence Assessment Tool for Psychiatric Advance Directives; GAF = Global Assessment of Functioning; ITAQ = Insight and Treatment Attitudes Questionnaire; HAM-D = Hamilton Depression Rating Scale; MacCAT–T = MacArthur Competence Assessment Tool for Treatment; MacCAT-CR = MacArthur Competence Assessment Tool for Clinical Research; PANSS = Positive and Negative Syndrome Scale; PSAS = Psychiatric Symptoms Assessment Scale; PSS = Problem Severity Summary; SAI–E = Expanded Schedule for Assessment of Insight; MMSE = Mini Mental State Examination; WAIS = Wechsler Adult Intelligence Scale.

**Table 3:** Studies on decisional capacity to consent to treatment, research, and advance directives.

Author	Country	Stage of Illness	Mean Age (Years) (SD)	% F	Time with Illness (Years)	Education (Years) (SD)
Srebnik et al., 2004 [8]	USA	Chronic	41.9 (9.3)	53	NA	NA
Cairns et al., 2005 [35]	UK	Acute	37.2 (11.8)	33.6	13.8	NA
Appelbaum & Redlich 2006 [34]	USA	Chronic	44.6 (10.0)	NA	NA	12.19 (2.4)
Palmer et al., 2007 [26]	USA	Chronic	54 (8.7)	50	24.2	13.3 (2.0)
López-Jaramillo et al., 2016 [36]	Colombia	Chronic	40.6 (11.4)	38.7	15.6	9.7 (10.8)
Mandarelli et al., 2018 [37]	Italy	Acute	39.8 (12.0)	37	7.3	11.3 (3.7)

**Table 4:** Understanding.

**Study**	**No. of BD**	**No. of SZ**	**ES**	**Sig.**	**SE**	**W**	**95% CI**	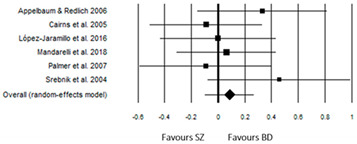
Appelbaum & Redlich 2006 [34]	22	63	0.33	0.180	0.25	14.21%	−0.15, 0.81	
Cairns et al., 2005 [35]	29	84	−0.09	0.680	0.21	18.88%	−0.51, 0.33	
López-Jaramillo et al., 2016 [36]	40	40	0.0	1.000	0.22	17.63%	−0.43, 0.43	
Mandarelli et al., 2018 [37]	47	65	0.06	0.735	0.19	23.90%	−0.31, 0.44	
Palmer et al., 2007 [26]	31	31	−0.09	0.708	0.25	13.73%	−0.59, 0.40	
Srebnik et al., 2004 [8]	20	41	0.46	0.091	0.27	11.65%	−0.07, 0.99	
Overall (random-effects model)	189	324	0.09	0.352	0.09	100%	−0.10, 0.27	

Heterogeneity: Q = 4.21; df = 5 (*p* = 0.519); I^2^ = 0%; Tau^2^ = 0.00.

**Table 5:** Appreciation.

**Study**	**No. of BD**	**No. of SZ**	**ES**	**Sig.**	**SE**	**W**	**95% CI**	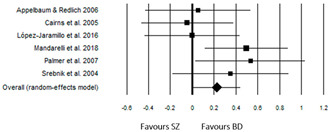
Appelbaum & Redlich 2006 [34]	22	63	0.05	0.823	0.25	15.15%	−0.43, 0.54	
Cairns et al., 2005 [35]	29	84	−0.04	0.840	0.21	18.57%	−0.46, 0.38	
López-Jaramillo et al., 2016 [36]	40	40	0.0	1.000	0.22	17.67%	−0.43, 0.43	
Mandarelli et al., 2018 [37]	47	65	0.50	0.010	0.19	21.41%	0.12, 0.87	
Palmer et al., 2007 [26]	31	31	0.53	0.037	0.26	14.25%	0.03, 1.03	
Srebnik et al., 2004 [8]	20	41	0.35	0.195	0.27	12.96%	−0.18, 0.88	
Overall (random-effects model)	189	324	0.23	0.037	0.11	100%	0.01, 0.44	

Heterogeneity: Q = 6.73; df = 5 (*p* = 0.242); I^2^ = 25.70%; Tau^2^ = 0.02.

**Table 6:** Reasoning.

**Study**	**No. of BD**	**No. of SZ**	**ES**	**Sig.**	**SE**	**W**	**95% CI**	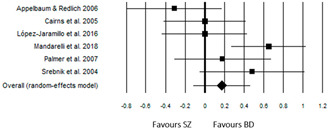
Appelbaum & Redlich 2006 [34]	22	63	−0.31	0.215	0.25	15.87%	−0.79, 0.18	
Cairns et al., 2005 [35]	29	84	0.00	0.985	0.21	17.79%	−0.05, 1.02	
López-Jaramillo et al., 2016 [36]	40	40	0.00	1.000	0.22	17.33%	−0.43, 0.43	
Mandarelli et al., 2018 [37]	47	65	0.65	0.001	0.20	18.96%	0.27, 1.04	
Palmer et al., 2007 [26]	31	31	0.18	0.462	0.25	15.60%	−0.31, 0.68	
Srebnik et al., 2004 [8]	20	41	0.49	0.074	0.27	14.45%	−0.05, 1.02	
Overall (random-effects model)	189	324	0.18	0.074	0.27	100%	−0.12, 0.47	

Heterogeneity: Q = 12.40; df = 5 (*p* = 0.030); I^2^ = 59.69%; Tau^2^ = 0.08.

**Table 7:** Expression of a choice.

**Study**	**No. of BD**	**No. of SZ**	**ES**	**Sig.**	**SE**	**W**	**95% CI**	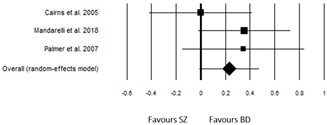
Cairns et al., 2005 [35]	29	84	0.00	1.000	0.21	33.76%	−0.42, 0.42	
Mandarelli et al., 2018 [37]	47	65	0.35	0.064	0.19	63.50%	−0.02, 0.73	
Palmer et al., 2007 [26]	31	31	0.35	0.167	0.25	36.50%	−0.15, 0.84	
Overall (random-effects model)	107	180	0.23	0.060	0.12	100%	−0.01, 0.48	

Heterogeneity: Q = 1.80; df = 2 (*p* = 0.407); I^2^ = 0.00%; Tau^2^ = 0.00.

## Data Availability

Not applicable.

## Supplementary Materials

The following supporting information can be downloaded at: https://www.mdpi.com/article/10.3390/medicina60050764/s1, Supplement S1: Lists of studies not included due to lack of useful data; Table S2: Studies employing similar instruments, such as MacCAT-T and Mac-CAT-CR; Table S3: Studies enrolling patients in a clinical remission status. References [59,60,61,62,63,64] are cited in Supplementary Materials.

## References

[1] Mandarelli G., Tarsitani L., Parmigiani G., Polselli G.M., Frati P., Biondi M., Ferracuti S. (2014). Mental capacity in patients involuntarily or voluntarily receiving psychiatric treatment for an acute mental disorder. J. Forensic Sci..

[2] Santurro A., Vullo A.M., Borro M., Gentile G., Russa R.L., Simmaco M., Frati P., Fineschi V. (2017). Personalized medicine applied to forensic sciences: New advances and perspectives for a tailored forensic approach. Curr. Pharm. Biotechnol..

[3] Turillazzi E., Neri M., Riezzo I., Frati P., Fineschi V. (2014). Informed consent in Italy-traditional versus the law: A gordian knot. Aesthetic Plast. Surg..

[4] Curley A., Watson C., Kelly B.D. (2022). Capacity to consent to treatment in psychiatry inpatients—A systematic review. Int. J. Psychiatry Clin. Pract..

[5] Lamont S., Jeon Y.-H., Chiarella M. (2013). Assessing patient capacity to consent to treatment: An integrative review of instruments and tools. J. Clin. Nurs..

[6] Kreutzer J.S., DeLuca J., Caplan B. (2011). MacArthur Competence Assessment Tool for Clinical Research (MacCAT-CR). Encyclopedia of Clinical Neuropsychology.

[7] Wong J. (2002). *MacCAT-CR: MacArthur Competence Tool for Clinical Research.* By P.S. Appelbaum and T. Grisso. (Pp. 84; $22.00.) Professional Resource Press: Sarasota, FL. 2001. Psychol. Med..

[8] Srebnik D., Appelbaum P.S., Russo J. (2004). Assessing competence to complete psychiatric advance directives with the competence assessment tool for psychiatric advance directives. Compr. Psychiatry.

[9] Appelbaum P.S. (2010). Consent in impaired populations. Curr. Neurol. Neurosci. Rep..

[10] Moye J., Karel M.J., Edelstein B., Hicken B., Armesto J.C., Gurrera R.J. (2007). Assessment of Capacity to Consent to Treatment. Clin. Gerontol..

[11] Carroll D.W. (2010). Assessment of Capacity for Medical Decision Making. J. Gerontol. Nurs..

[12] Buchanan A. (2004). Mental capacity, legal competence and consent to treatment. J. R. Soc. Med..

[13] Tannou T., Koeberlé S., Aubry R., Haffen E. (2020). How does decisional capacity evolve with normal cognitive aging: Systematic review of the literature. Eur. Geriatr. Med..

[14] Stroup S., Appelbaum P., Swartz M., Patel M., Davis S., Jeste D., Kim S., Keefe R., Manschreck T., McEvoy J. (2005). Decision-making capacity for research participation among individuals in the CATIE schizophrenia trial. Schizophr. Res..

[15] Calcedo-Barba A., Fructuoso A., Martinez-Raga J., Paz S., Sánchez de Carmona M., Vicens E. (2020). A meta-review of literature reviews assessing the capacity of patients with severe mental disorders to make decisions about their healthcare. BMC Psychiatry.

[16] Lepping P., Stanly T., Turner J. (2015). Systematic review on the prevalence of lack of capacity in medical and psychiatric settings. Clin. Med..

[17] Gupta U.C., Kharawala S. (2012). Informed consent in psychiatry clinical research: A conceptual review of issues, challenges, and recommendations. Perspect. Clin. Res..

[18] Jeste D.V., Depp C.A., Palmer B.W. (2006). Magnitude of Impairment in Decisional Capacity in People With Schizophrenia Compared to Normal Subjects: An Overview. Schizophr. Bull..

[19] Misra S., Socherman R., Park B.S., Hauser P., Ganzini L. (2008). Influence of mood state on capacity to consent to research in patients with bipolar disorder. Bipolar Disord..

[20] Spencer B.W.J., Shields G., Gergel T., Hotopf M., Owen G.S. (2017). Diversity or disarray? A systematic review of decision-making capacity for treatment and research in schizophrenia and other non-affective psychoses. Psychol. Med..

[21] Wang S.-B., Wang Y.-Y., Ungvari G.S., Ng C.H., Wu R.-R., Wang J., Xiang Y.-T. (2017). The MacArthur Competence Assessment Tools for assessing decision-making capacity in schizophrenia: A meta-analysis. Schizophr. Res..

[22] Spencer BW J., Gergel T., Hotopf M., Owen G.S. (2018). Unwell in hospital but not incapable: Cross-sectional study on the dissociation of decision-making capacity for treatment and research in in-patients with schizophrenia and related psychoses. Br. J. Psychiatry.

[23] Hostiuc S., Rusu M.C., Negoi I., Drima E. (2018). Testing decision-making competency of schizophrenia participants in clinical trials. A meta-analysis and meta-regression. BMC Psychiatry.

[24] Director S. (2023). Bipolar disorder and competence. J. Med. Ethics.

[25] Klein C.C., Jolson M.B., Lazarus M., Masterson B., Blom T.J., Adler C.M., DelBello M.P., Strakowski S.M. (2019). Capacity to provide informed consent among adults with bipolar disorder. J. Affect. Disord..

[26] Palmer B.W., Dunn L.B., Depp C.A., Eyler L.T., Jeste D.V. (2007). Decisional capacity to consent to research among patients with bipolar disorder: Comparison with schizophrenia patients and healthy subjects. J. Clin. Psychiatry.

[27] Cohen B.J., McGarvey E.L., Pinkerton R.C., Kryzhanivska L. (2004). Willingness and competence of depressed and schizophrenic inpatients to consent to research. J. Am. Acad. Psychiatry Law.

[28] Hindmarch T., Hotopf M., Owen G.S. (2013). Depression and decision-making capacity for treatment or research: A systematic review. BMC Med. Ethics.

[29] Koukopoulos A., Mandarelli G., Maglio G., Macellaro M., Cifrodelli M., Kotzalidis G., Tarsitani L., Biondi M., Ferracuti S. (2020). Evaluation of the capacity to consent to treatment among patients with bipolar disorder: Comparison between the acute psychopathological episode and the stable mood phase. J. Affect. Disord. Rep..

[30] Liberati A., Altman D.G., Tetzlaff J., Mulrow C., Gøtzsche P.C., Ioannidis J.P.A., Clarke M., Devereaux P.J., Kleijnen J., Moher D. (2009). The PRISMA statement for reporting systematic reviews and meta-analyses of studies that evaluate health care interventions: Explanation and elaboration. J. Clin. Epidemiol..

[31] Page M.J., McKenzie J.E., Bossuyt P.M., Boutron I., Hoffmann T.C., Mulrow C.D., Shamseer L., Tetzlaff J.M., Akl E.A., Brennan S.E. (2021). The PRISMA 2020 statement: An updated guideline for reporting systematic reviews. BMJ (Clin. Res. Ed.).

[32] Moskalewicz A., Oremus M. (2020). No clear choice between Newcastle-Ottawa Scale and Appraisal Tool for Cross-Sectional Studies to assess methodological quality in cross-sectional studies of health-related quality of life and breast cancer. J. Clin. Epidemiol..

[33] Wells G., Shea B., O’Connell D., Peterson J., Welch V., Losos M., Tugwell P. (2000). The Newcastle-Ottawa Scale (NOS) for Assessing the Quality of Nonrandomised Studies in Meta-Analyses.

[34] Appelbaum P.S., Redlich A. (2006). Impact of decisional capacity on the use of leverage to encourage treatment adherence. Community Ment. Health J..

[35] Cairns R., Maddock C., Buchanan A., David A.S., Hayward P., Richardson G., Szmukler G., Hotopf M. (2005). Prevalence and predictors of mental incapacity in psychiatric in-patients. Br. J. Psychiatry J. Ment. Sci..

[36] López-Jaramillo C., Tobler C.A., Gómez C.O., Triana J.E. (2016). Correlation Between Insight and Capacity to Consent to Research in Subjects With Bipolar Disorder Type I and Schizophrenia. Rev. Colomb. Psiquiatr..

[37] Mandarelli G., Carabellese F., Parmigiani G., Bernardini F., Pauselli L., Quartesan R., Catanesi R., Ferracuti S. (2018). Treatment decision-making capacity in non-consensual psychiatric treatment: A multicentre study. Epidemiol. Psychiatr. Sci..

[38] Overall J.E., Gorham D.R. (1962). The Brief Psychiatric Rating Scale. Psychol. Rep..

[39] Crocetti E. (2016). Systematic Reviews With Meta-Analysis: Why, When, and How?. Emerg. Adulthood.

[40] Brydges C.R. (2019). Effect Size Guidelines, Sample Size Calculations, and Statistical Power in Gerontology. Innov. Aging.

[41] Egger M., Davey Smith G., Schneider M., Minder C. (1997). Bias in meta-analysis detected by a simple, graphical test. BMJ Clin. Res. Ed..

[42] Grisso T., Appelbaum P.S., Hill-Fotouhi C. (1997). The MacCAT-T: A clinical tool to assess patients’ capacities to make treatment decisions. Psychiatr. Serv..

[43] Hirakawa H. (2023). Assessing Medical Decision-Making Competence Using the MacArthur Competence Assessment Tool-Treatment for Schizophrenia. Prim. Care Companion CNS Disord..

[44] Owen G.S., David A.S., Richardson G., Szmukler G., Hayward P., Hotopf M. (2009). Mental capacity, diagnosis and insight in psychiatric in-patients: A cross-sectional study. Psychol. Med..

[45] Raffard S., Lebrun C., Laraki Y., Capdevielle D. (2021). Validation of the French Version of the MacArthur Competence Assessment Tool for Treatment (MacCAT-T) in a French Sample of Individuals with Schizophrenia: Validation de la version française de l’instrument d’évaluation des compétences MacArthur-traitement (MacCAT-T) dans un échantillon français de personnes souffrant de schizophrénie. Can. J. Psychiatry. Rev. Can. Psychiatr..

[46] Capdevielle D., Raffard S., Bayard S., Garcia F., Baciu O., Bouzigues I., Boulenger J.-P. (2009). Competence to consent and insight in schizophrenia: Is there an association? A pilot study. Schizophr. Res..

[47] Belmaker R.H., Bersudsky Y. (2009). Bipolar Disorder: Mania and Depression. Discov. Med..

[48] Sanchez-Moreno J., Martinez-Aran A., Tabarés-Seisdedos R., Torrent C., Vieta E., Ayuso-Mateos J.L. (2009). Functioning and disability in bipolar disorder: An extensive review. Psychother. Psychosom..

[49] Thomas S.P., Nisha A., Varghese P.J. (2016). Disability and Quality of Life of Subjects with Bipolar Affective Disorder in Remission. Indian J. Psychol. Med..

[50] Chen M., Fitzgerald H.M., Madera J.J., Tohen M. (2019). Functional outcome assessment in bipolar disorder: A systematic literature review. Bipolar Disord..

[51] Vaskinn A., Haatveit B., Melle I., Andreassen O.A., Ueland T., Sundet K. (2020). Cognitive Heterogeneity across Schizophrenia and Bipolar Disorder: A Cluster Analysis of Intellectual Trajectories. J. Int. Neuropsychol. Soc. JINS.

[52] Carpenter W.T., Gold J.M., Lahti A.C., Queern C.A., Conley R.R., Bartko J.J., Kovnick J., Appelbaum P.S. (2000). Decisional capacity for informed consent in schizophrenia research. Arch. Gen. Psychiatry.

[53] Moser D.J., Schultz S.K., Arndt S., Benjamin M.L., Fleming F.W., Brems C.S., Paulsen J.S., Appelbaum P.S., Andreasen N.C. (2002). Capacity to provide informed consent for participation in schizophrenia and HIV research. Am. J. Psychiatry.

[54] Di Fazio N., Morena D., Piras F., Piras F., Banaj N., Delogu G., Damato F., Frati P., Fineschi V., Ferracuti S. (2024). Reliability of clinical judgment for evaluation of informed consent in mental health settings and the validation of the Evaluation of Informed Consent to Treatment (EICT) scale. Front. Psychol..

[55] Vöhringer P.A., Barroilhet S.A., Amerio A., Reale M.L., Alvear K., Vergne D., Ghaemi S.N. (2013). Cognitive impairment in bipolar disorder and schizophrenia: A systematic review. Front. Psychiatry.

[56] McIntyre R.S., Berk M., Brietzke E., Goldstein B.I., López-Jaramillo C., Kessing L.V., Malhi G.S., Nierenberg A.A., Rosenblat J.D., Majeed A. (2020). Bipolar disorders. Lancet.

[57] American Psychiatric Association (2013). Diagnostic and Statistical Manual of Mental Disorders (DSM-5®).

[58] Gowensmith W.N., Murrie D.C., Boccaccini M.T. (2013). How reliable are forensic evaluations of legal sanity?. Law Hum. Behav..

[59] Carabellese F., Mandarelli G., La Tegola D., Parmigiani G., Ferracuti S., Quartesan R., Bellomo A., Catanesi R. (2017). Mental capacity e capacity to consent: Studio multicentrico in un campione di pazienti ricoverati in TSO [Mental capacity and capacity to consent: Multicentric study in a involuntary psychiatric hospitalized patients sample]. Riv. Psichiatr..

[60] Fernandez C., Kennedy H.G., Kennedy M. (2017). The recovery of factors associated with decision-making capacity in individuals with psychosis. BJPsych Open.

[61] Howe V., Foister K., Jenkins K., Skene L., Copolov D., Keks N. (2005). Competence to give informed consent in acute psychosis is associated with symptoms rather than diagnosis. Schizophr. Res..

[62] Morán-Sánchez I., Luna A., Pérez-Cárceles M.D. (2016). Assessment of Capacity to Consent to Research Among Psychiatric Outpatients: Prevalence and Associated Factors. Psychiatr. Q..

[63] Palmer B.W., Savla G.N., Roesch S.C., Jeste D.V. (2013). Changes in capacity to consent over time in patients involved in psychiatric research. Br. J. Psychiatry.

[64] Tinland A., Loubière S., Mougeot F., Jouet E., Pontier M., Baumstarck K., Loundou A., Franck N., Lançon C., Auquier P. (2022). Effect of Psychiatric Advance Directives Facilitated by Peer Workers on Compulsory Admission Among People With Mental Illness. JAMA Psychiatry.

