# Supplementary material for: Capacity to Consent in Healthcare: A Systematic Review and Meta-Analysis Comparing Patients with Bipolar Disorders and Schizophrenia Spectrum Disorders
Source: Medicina (Kaunas). 2024 May 5;60(5):764. doi: 10.3390/medicina60050764 (PMC11123007; doi:10.3390/medicina60050764)
Supplement: Supplementary file 1 [file medicina-60-00764-s001.zip › Supplementary material S1.pdf]

Supplement S1 - Lists of studies not included due to lack of useful data

1. Carabellese, F., Mandarelli, G., La Tegola, D., Parmigiani, G., Ferracuti, S., Quartesan, R., Bellomo, A., & Catanesi, R. (2017). Mental capacity e capacity to consent: studio multicentrico in un campione di pazienti ricoverati in TSO [Mental capacity and capacity to consent: multicentric study in a involuntary psychiatric hospitalized patients sample]. *Rivista di psichiatria*, *52*(2), 67–74. https://doi.org/10.1708/2679.27442
2. Fernandez, C., Kennedy, H. G., & Kennedy, M. (2017). The recovery of factors associated with decision-making capacity in individuals with psychosis. BJPsych open, 3(3), 113–119. https://doi.org/10.1192/bjpo.bp.116.004226
3. Howe, V., Foister, K., Jenkins, K., Skene, L., Copolov, D., & Keks, N. (2005). Competence to give informed consent in acute psychosis is associated with symptoms rather than diagnosis. *Schizophrenia research*, *77*(2-3), 211–214. https://doi.org/10.1016/j.schres.2005.03.005
4. Mandarelli, G., Tarsitani, L., Parmigiani, G., Polselli, G. M., Frati, P., Biondi, M., & Ferracuti, S. (2014). Mental capacity in patients involuntarily or voluntarily receiving psychiatric treatment for an acute mental disorder. *Journal of forensic sciences*, *59*(4), 1002–1007. https://doi.org/10.1111/1556-4029.12420
5. Morán-Sánchez, I., Luna, A., & Pérez-Cárceles, M. D. (2016). Assessment of Capacity to Consent to Research Among Psychiatric Outpatients: Prevalence and Associated Factors. The Psychiatric quarterly, 87(1), 89–105. https://doi.org/10.1007/s11126-015-9365-3
6. Owen, G. S., David, A. S., Richardson, G., Szmukler, G., Hayward, P., & Hotopf, M. (2009). Mental capacity, diagnosis and insight in psychiatric in-patients: a cross-sectional study. *Psychological medicine*, *39*(8), 1389–1398. https://doi.org/10.1017/S0033291708004637
7. Palmer, B. W., Savla, G. N., Roesch, S. C., & Jeste, D. V. (2013). Changes in capacity to consent over time in patients involved in psychiatric research. *The British journal of psychiatry : the journal of mental science*, *202*(6), 454–458. https://doi.org/10.1192/bjp.bp.112.121160
8. Tinland, A., Loubière, S., Mougeot, F., Jouet, E., Pontier, M., Baumstarck, K., Loundou, A., Franck, N., Lançon, C., Auquier, P., & DAiP Group (2022). Effect of Psychiatric Advance Directives Facilitated by Peer Workers on Compulsory Admission Among People With Mental Illness: A Randomized Clinical Trial. *JAMA psychiatry*, *79*(8), 752–759. https://doi.org/10.1001/jamapsychiatry.2022.1627
