# Supplementary material for: Capacity to Consent in Healthcare: A Systematic Review and Meta-Analysis Comparing Patients with Bipolar Disorders and Schizophrenia Spectrum Disorders
Source: Medicina (Kaunas). 2024 May 5;60(5):764. doi: 10.3390/medicina60050764 (PMC11123007; doi:10.3390/medicina60050764)

Tables S2

Studies employing similar instruments, such as MacCAT-T and Mac-CAT-CR

Understanding

Study	No. of BD	No. of SZ	ES	Sig.	SE	W	95% CI
Appelbaum & Redlich 2006	22	63	0.33	0.180	0.25	16.08 %	- 0.15, 0.81
Cairns et al. 2005	29	84	-0.09	0.680	0.21	21.37	- 0.51, 0.33
López-Jaramillo et al. 2016	40	40	0.00	1.000	0.22	19.96	- 0.43, 0.43
Mandarelli et al. 2018	47	65	0.06	0.735	0.19	27.05	- 0.31, 0.44
Palmer et al. 2007	31	31	-0.09	0.708	0.25	15.54	- 0.59, 0.40
Overall (random-effects model)	169	283	0.04	0.707	0.10	100 %	- 0.16, 0.23
Heterogeneity: Q=2.08; df = 4 (p = 0.720); $I^2 = 0.00$; $Tau^2 = 0.00$							

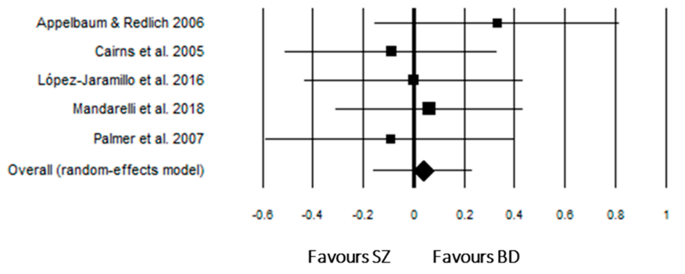

Appreciation

Study	No. of BD	No. of SZ	ES	Sig.	SE	W	95% CI
Appelbaum & Redlich 2006	22	63	0.05	0.823	0.25	17.82	- 0.43, 0.54
Cairns et al. 2005	29	84	- 0.04	0.840	0.21	21.17	- 0.46, 0.38
López-Jaramillo et al. 2016	40	40	0.00	1.000	0.22	20.31	- 0.43, 0.43
Mandarelli et al. 2018	47	65	0.50	0.010	0.19	23.80	0.12, 0.87
Palmer et al. 2007	31	31	0.53	0.037	0.26	16.90	0.03, 1.03
Overall (random-effects model)	169	283	0.21	0.102	0.13	100 %	- 0.04, 0.46
Heterogeneity: Q=6.50; df = 4 (p = 0.165); I² = 38.43%; Tau² = 0.03							

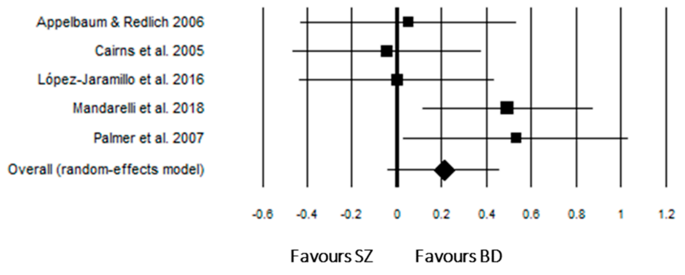

Reasoning

Study	No. of BD	No. of SZ	ES	Sig.	SE	W	95% CI
Appelbaum & Redlich 2006	22	63	- 0.31	0.215	0.25	18.65	- 0.79, 0.18
Cairns et al. 2005	29	84	0.00	0.985	0.21	20.74	- 0.42, 0.42
López-Jaramillo et al. 2016	40	40	0.00	1.000	0.22	20.25	- 0.43, 0.43
Mandarelli et al. 2018	47	65	0.65	0.001	0.20	22.00	0.27, 1.04
Palmer et al. 2007	31	31	0.18	0.462	0.25	18.36	- 0.31, 0.68
Overall (random-effects model)	169	283	0.12	0.465	0.17	100 %	- 0.21, 0.45
Heterogeneity: Q=11.05; df = 4 (p = 0.465); I² = 63.81%; Tau² = 0.09							

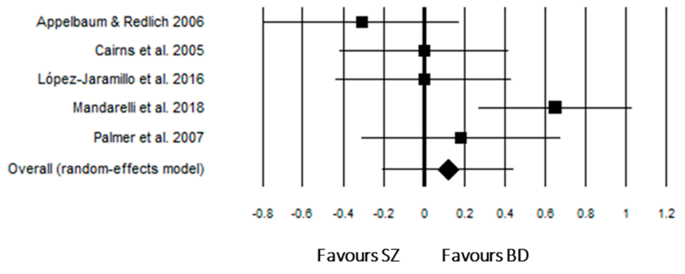

Supplement: Supplementary file 1 [file medicina-60-00764-s001.zip › Supplementary material S2.pdf]
