# Supplementary material for: Capacity to Consent in Healthcare: A Systematic Review and Meta-Analysis Comparing Patients with Bipolar Disorders and Schizophrenia Spectrum Disorders
Source: Medicina (Kaunas). 2024 May 5;60(5):764. doi: 10.3390/medicina60050764 (PMC11123007; doi:10.3390/medicina60050764)

Tables S3

Studies enrolling patients in a clinical remission status

Understanding

Study	No. of BD	No. of SZ	ES	Sig.	SE	W	95% CI
Appelbaum & Redlich 2006	22	63	0.33	0.180	0.25	24.88	- 0.15, 0.81
López-Jaramillo et al. 2016	40	40	0.00	1.000	0.22	30.34	- 0.43, 0.43
Palmer et al. 2007	31	31	-0.09	0.708	0.25	24.11	- 0.59, 0.40
Srebnik et al. 2004	20	41	0.46	0.091	0.27	20.67	- 0.07, 0.99
Overall (random-effects model)	113	175	0.15	0.226	0.13	100 %	- 0.10, 0.41
Heterogeneity: Q=3.24; df = 3 (p = 0.356); I^2 = 7.37; Tau^2 = 0.00							

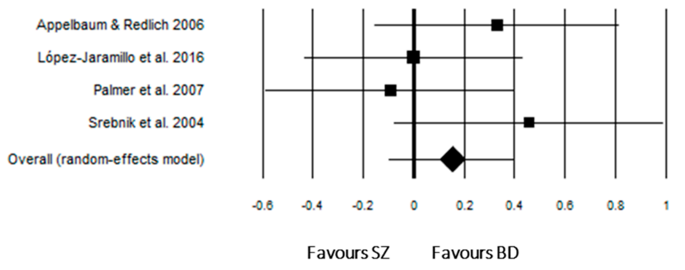

Appreciation

Study	No. of BD	No. of SZ	ES	Sig.	SE	W	95% CI
Appelbaum & Redlich 2006	22	63	0.05	0.823	0.25	25.20 %	- 0.43, 0.54
López-Jaramillo et al. 2016	40	40	0.00	1.000	0.22	30.58 %	- 0.43, 0.43
Palmer et al. 2007	31	31	0.53	0.037	0.26	23.38 %	0.03, 1.03
Srebnik et al. 2004	20	41	0.35	0.195	0.27	20.85 %	- 0.18, 0.88
Overall (random-effects model)	113	175	0.21	0.095	0.13	100 %	- 0.04, 0.46
Heterogeneity: Q=3.17; df = 3 (p = 0.367); I² = 5.27%; Tau² = 0.00							

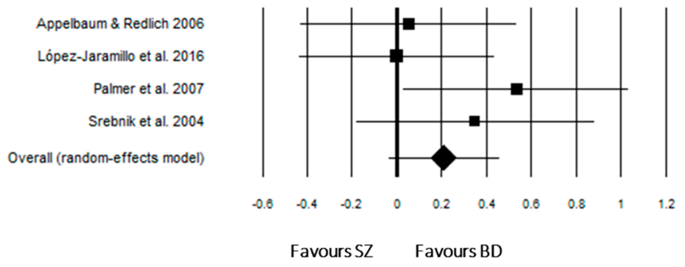

Reasoning

Study	No. of BD	No. of SZ	ES	Sig.	SE	W	95% CI
Appelbaum & Redlich 2006	22	63	- 0.31	0.215	0.25	25.07 %	- 0.79, 0.18
López-Jaramillo et al. 2016	40	40	0.00	1.000	0.22	28.36 %	- 0.43, 0.43
Palmer et al. 2007	31	31	0.18	0.462	0.25	24.49 %	- 0.31, 0.68
Srebnik et al. 2004	20	41	0.49	0.074	0.27	22.08 %	- 0.05, 1.02
Overall (random-effects model)	113	175	0.08	0.632	0.16	100 %	- 0.24, 0.39
Heterogeneity: Q=4.98; df = 3 (p = 0.173); I² = 39.79%; Tau² = 0.04							

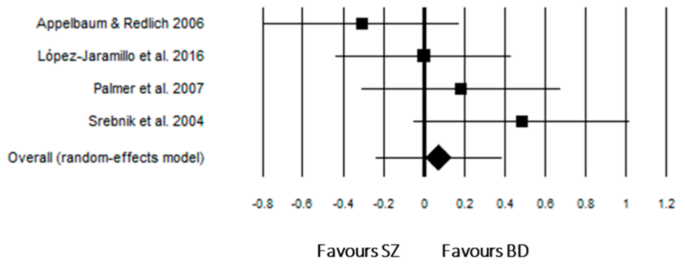

Expression of a Choice

Study	No. of BD	No. of SZ	ES	Sig.	SE	95% CI
Palmer et al. 2007	31	31	0.35	0.167	0.25	- 0.15, 0.84

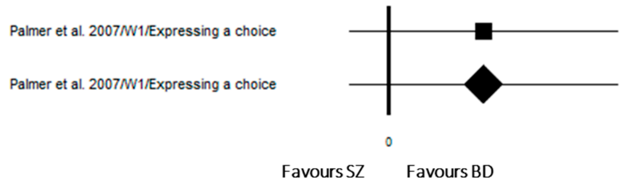

Supplement: Supplementary file 1 [file medicina-60-00764-s001.zip › Supplementary material S3.pdf]
